# Convergences and Divergences of Thymus- and Peripherally Derived Regulatory T Cells in Cancer

**DOI:** 10.3389/fimmu.2013.00247

**Published:** 2013-08-27

**Authors:** Alessia Burocchi, Mario P. Colombo, Silvia Piconese

**Affiliations:** ^1^Molecular Immunology Unit, Department of Experimental Medicine, Fondazione IRCCS “Istituto Nazionale Tumori,”Milan, Italy; ^2^Cellular and Molecular Immunology Unit, Dipartimento di Medicina Interna e Specialità Mediche, “Sapienza” Università di Roma, Rome, Italy

**Keywords:** T_reg_ development, heterogeneity, specialization, plasticity, epigenetic commitment, tumor antigens

## Abstract

The expansion of regulatory T cells (T_reg_) is a common event characterizing the vast majority of human and experimental tumors and it is now well established that T_reg_ represent a crucial hurdle for a successful immunotherapy. T_reg_ are currently classified, according to their origin, into thymus-derived T_reg_ (tT_reg_) or peripherally induced T_reg_ (pT_reg_) cells. Controversy exists over the prevalent mechanism accounting for T_reg_ expansion in tumors, since both tT_reg_ proliferation and *de novo* pT_reg_ differentiation may occur. Since tT_reg_ and pT_reg_ are believed as preferentially self-specific or broadly directed to non-self and tumor-specific antigens, respectively, the balance between tT_reg_ and pT_reg_ accumulation may impact on the repertoire of antigen specificities recognized by T_reg_ in tumors. The prevalence of tT_reg_ or pT_reg_ may also affect the outcome of immunotherapies based on tumor-antigen vaccination or T_reg_ depletion. The mechanisms dictating pT_reg_ induction or tT_reg_ expansion/stability are a matter of intense investigation and the most recent results depict a complex landscape. Indeed, selected T_reg_ subsets may display peculiar characteristics in terms of stability, suppressive function, and cytokine production, depending on microenvironmental signals. These features may be differentially distributed between pT_reg_ and tT_reg_ and may significantly affect the possibility of manipulating T_reg_ in cancer therapy. We propose here that innovative immunotherapeutic strategies may be directed at diverting unstable/uncommitted T_reg_, mostly enriched in the pT_reg_ pool, into tumor-specific effectors, while preserving systemic immune tolerance ensured by self-specific tT_reg_.

## T_reg_ Suppress Pro-Tumoral Inflammation or Anti-Tumor Response

### Introduction

Tumor onset is a very complex process, in which cells of both innate and adaptive immune system play crucial roles in inhibiting or fostering tumor development. The awareness that the immune system could act as an extrinsic tumor suppressor or as a tumor-sculpting player resulted in the cancer immunoediting theory, which described the interaction between tumor and host as consisting of three different phases: elimination, equilibrium, and escape (1). During the last phase of this process, transformed cells acquire the ability to subvert the control exerted by immune cells thus originating the clinically evident pathology. The escape is due to different mechanisms, including reduced immunogenicity (low expression level of MHC class I and loss of antigen expression), acquired resistance to the cytotoxic functions of immune cells, and accumulation in the tumor microenvironment of immunosuppressive mediators, like regulatory T cells (T_reg_) (1). The first marker to be identified as distinguishing T_reg_ from the other CD4^+^ T lymphocytes was CD25 (2) and depletion of CD25-positive cells unveiled anti-tumor immunity in experimental models (3). Few years later, the transcription factor Forkhead Box P3 (Foxp3) was indicated as the master regulator of T_reg_ (4, 5). In support of the crucial roles played by Foxp3 in T_reg_ fate determination and immune homeostasis, Foxp3 mutations have been recognized as responsible for human Immune Dysfunction, Polyendocrinopathy, Enteropathy, X-linked (IPEX) syndrome (6, 7), and for the phenotype of scurfy mutant mice (8), both characterized by fatal autoimmune lymphoproliferation linked to severe defects in T_reg_ development/functions. However, very recent data have demonstrated that the complete development of the T_reg_ lineage requires the concomitant, Foxp3-independent, establishment of a T_reg_-specific pattern of DNA hypomethylation (9).

According to recently proposed recommendations (10), T_reg_ are classified into two principal subsets based on their developmental origin: thymus-derived T_reg_ (tT_reg_) develop in the thymus, while peripherally induced T_reg_ (pT_reg_) develop *in vivo* in the periphery from conventional T cells (T_conv_), through a process called “conversion” (11). T_reg_ can also be induced *in vitro* (and are called iT_reg_) under TGF-β and/or retinoic acid exposure, but their complete commitment into fully differentiated T_reg_ is still under debate (12). In physiological conditions, the pool of T_reg_, encompassing both tT_reg_ and pT_reg_, which represents about the 5–10% of the circulating CD4^+^ T lymphocytes, assures peripheral self-tolerance and prevents exacerbated immune responses (7, 8). A huge amount of data now demonstrates that tumor onset and progression perturb T_reg_ homeostasis and lead to increased T_reg_/T_conv_ and T_reg_/CD8 ratios both in peripheral blood and in the tumor microenvironment (13). The accumulation of T_reg_ at tumor sites may be due to the concomitant or the preferential occurrence of distinct events, such as the recruitment of T_reg_ from periphery, the proliferation of pre-existing T_reg_ in the tumor microenvironment, and the *de novo* conversion of tumor-infiltrating CD4^+^ lymphocytes (TIL) into pT_reg_ (14, 15). Despite controversy existing over the dominant suppression mechanism, and despite the incomplete understanding of the biological meaning of T_reg_ accumulation in cancer, it is well accepted that T_reg_ are crucial players in tumor biology and that the modulation of their function is an indispensable requisite for the development of successful anti-tumor immune-therapies.

### Mechanisms of T_reg_ suppression in tumors

**Figure 1:**
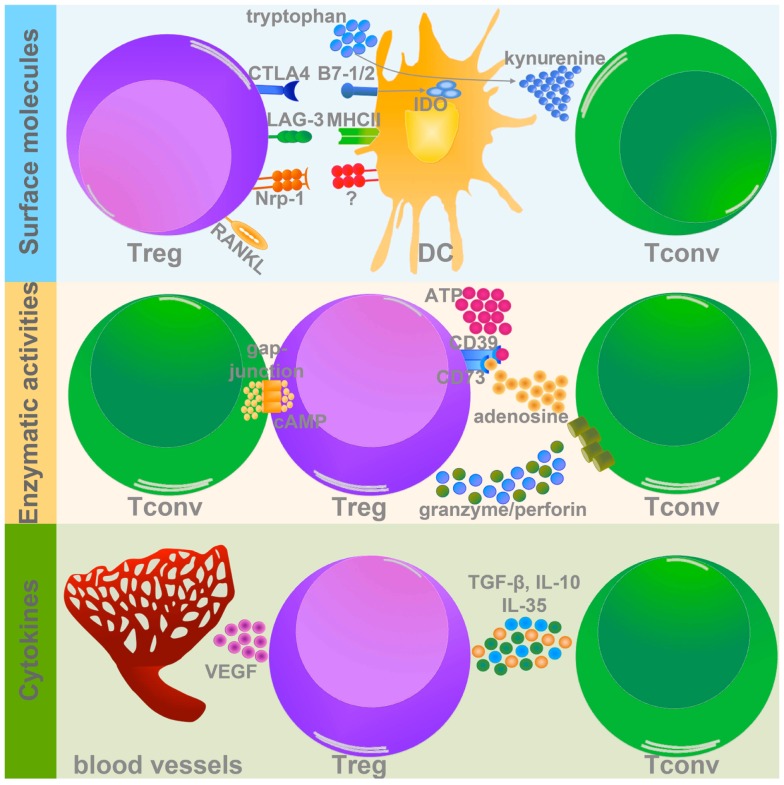
**T_reg_ suppressive mechanisms in tumor microenvironment**. T_reg_ use different strategies to inhibit target cells within the tumor mass. Three types of T_reg_-related molecules can mediate these suppressive mechanisms: (1) surface molecules (upper panel); (2) enzymes (middle panel), and (3) cytokines (lower panel). (1) Among the surface molecules expressed by T_reg_, CTLA-4, LAG-3, Nrp-1, and RANKL have a well-demonstrated role in promoting tumor progression, mainly modulating DC activation and function. In particular CTLA-4 and LAG-3, binding to CD80/CD86 (B7-1/2), and MHCII respectively, significantly impair DC capacity to activate T_conv_. In addition CTLA-4 promotes IDO expression and the production of the pro-apoptotic metabolite kynurenine. Nrp-1 instead stabilizes T_reg_-DC contact, allowing T_reg_ to adequately suppress DC. Although the course of action of RANKL is not yet well defined, its expression is associated to tumor metastatization. (2) The two ecto-enzymes CD39 and CD73 generate from ATP pericellular adenosine, which is endowed with strong tolerogenic effects. Also cAMP, similarly to adenosine, interferes with T_conv_ activation and survival. Granzyme and perforin induce the apoptosis of target cells by cytolysis. (3) T_reg_ secrete several immune-modulatory cytokines, which could directly modulate T_conv_ functions (TGF-β, IL-10, and IL-35), or indirectly promote the establishment of pro-tumorigenic microenvironment (VEGF).

It was recently demonstrated that T_reg_ infiltrating different tissues have a specific gene signature (16), thus T_reg_ may use peculiar suppression mechanisms in response to microenvironmental stimuli. This specialization may represent the basis for designing immune interventions targeting specific T_reg_ functions in a given tissue while sparing systemic immune homeostasis. Even though a tumor T_reg_-specific gene signature has not been delineated yet, some mechanisms have been described to contribute to T_reg_ suppression in tumors, which can be clustered in three main types: surface molecules, enzymatic activities, and cytokines (Figure 1).

Both human and mouse T_reg_ constitutively express on their surface cytotoxic T lymphocyte-associated antigen 4 (CTLA-4), a co-inhibitory member of the CD28/B7 family, endowed with strong immune-regulatory properties (17). The relevance of CTLA-4 in regulating T_reg_ function was demonstrated in several settings, including autoimmune diseases and different tumor types (18). A comparative gene expression profile between T_reg_ and T_conv_ revealed that T_reg_ specifically up-modulate lymphocyte activation gene 3 (LAG-3) (19), a homolog of CD4, that binds to MHC class II on antigen-presenting cells (APCs). LAG-3 is upregulated in tumor-infiltrating T_reg_ and experiments with anti-LAG-3 mAb demonstrated that functional LAG-3 is required for maximal T_reg_ suppressive function (20, 21). T_reg_-DC interaction is also mediated by the transmembrane protein neuropilin-1 (Nrp-1), expressed on T_reg_ membrane, which ensures the stability of T_reg_-DC interaction and allows T_reg_ to efficaciously suppress DC (22). A study conducted on patients with early-stage cervical tumor showed that T_reg_ infiltrating the tumor-draining lymph node of patients with metastasis have a higher expression of several immune-modulatory molecules, including Nrp-1 (23). The receptor activator of nuclear factor-kB ligand (RANKL), member of the tumor necrosis factor family, was found to be highly expressed on T_reg_ isolated from tumor-bearing hosts, and substantial evidence indicates that RANKL expressed by T_reg_ is involved in the onset of metastasis in both mammary (24) and prostate tumors (25).

Regulatory T cell suppression may be mediated by enzymatic activities, such as CD39/CD73 (26, 27), granzyme B, and perforin (28). CD39 and CD73 are two ecto-enzymes that dephosphorylate extracellular ATP and generate pericellular adenosine, which in turn exerts a strong pro-tumorigenic role modulating the function of numerous tumor-infiltrating immune cells. CD73-deficient mice develop a stronger anti-tumor immune response compared to CD73-sufficient mice (29). T_reg_ are also able to control the proliferation and function of different immune cells via the Perforin pathway (30). In mouse models of melanoma, lymphoma, and acute myeloid leukemia it has been demonstrated that tumor-infiltrating T_reg_, but not naïve T_reg_, secrete high amounts of both perforin and granzyme B, which in turn induce NK and CD8^+^ T cell death (28).

Immunosuppressive cytokines, like TGF-β and IL-10, are critical players in T_reg_ biology, being involved in both their differentiation and suppressive potential, especially in tumors. T_reg_-derived TGF-β was found relevant in suppression of anti-tumor T cell response in both mouse (31) and human (32, 33) tumors. IL-10 is a well-known immunosuppressive mediator, and several pieces of evidence highlight the relevance of T_reg_-derived IL-10 in controlling inflammation at the mucosal interfaces such as gut and lung (34, 35). Despite these data, little information is available about the roles of T_reg_-derived IL-10 in tumor microenvironments. We have recently demonstrated that tumor-associated T_reg_ secrete high amounts of IL-10, which in turn impairs DC migration to the draining lymph nodes and the mounting of a specific anti-tumor immune response. This phenotype could be reverted by stimulating the receptor OX40 on the surface of intratumoral T_reg_. Indeed, OX40-triggered T_reg_ showed reduced secretion of IL-10 as a consequence of the down-modulation of the interferon regulatory factor 1 (IRF1), a transcription factor active in the IL-10 promoter (36). Another cytokine recently described as critical for the full T_reg_ suppressive function is IL-35, formed by Epstein–Barr-virus-induced gene 3 (Ebi3) and IL-12α (p35) (37). In two different mouse transplantable tumor models (melanoma and colon carcinoma), it was observed that T_reg_ secrete abundant IL-35, thus promoting the differentiation of induced IL-35-secreting T_reg_ (37). It is well known that tumor growth is associated with a consistent process of new angiogenesis in response to hypoxia. A circuit involving tumor hypoxia, T_reg_ recruitment, and angiogenesis has been recently discovered (38). In the hypoxic tumor microenvironment, the chemokine axis CCL28–CCR10 plays a determinant role in the recruitment of T_reg_, which secrete huge amounts of vascular endothelial growth factor (VEGF), further stimulating the new angiogenesis process and the establishment of a tolerogenic microenvironment (38).

### Double effects of T_reg_ on prognosis

Since their discovery, T_reg_ were considered one of the main obstacles for tumor clearance, according to their tolerogenic properties and their accumulation along tumor progression. In this view, several anti-tumor immune-therapies focus on T_reg_ depletion or inhibition, in order to “contrasuppress” T_reg_ inhibitory functions and to block the conversion of non-regulatory cells (non-T_reg_) into regulatory cells (15). Reduced T_conv_/T_reg_ ratio was observed in patients with pancreatic tumor (39, 40), breast cancer (39), ovarian cancer (41), Hodgkin lymphoma (42), and melanoma (43). Increased T_reg_ frequency is generally associated to advanced tumor stage and poor prognosis, as recently demonstrated in a study on ovarian cancer (44). In the ascites of patients with advanced tumor, the percentage of T_reg_ was increased compared to the ascites of patients with early-stage tumor. Same results were obtained with the mouse WF-3 transplantable ovarian tumor model, showing augmented percentages of T_reg_ in both spleen and tumor-associated cells of mice with advanced tumors, compared to naïve or mice with early lesions. In addition, the treatment of tumor-bearing mice with the T_reg_-depleting mAb PC61 (αCD25) reduced tumor growth and prolonged mice survival (44). Similarly, it has been demonstrated that T_reg_ number inversely correlated with the therapy outcome in melanoma patients treated with non-myeloablative chemotherapy, in combination or not with total body irradiation, followed by adoptive T cell transfer (45). Responder patients had a lower frequency of T_reg_ in peripheral blood compared to non-responder patients (45). A study conducted on patients affected by invasive ductal carcinoma showed a positive correlation among T_reg_, Th17, and tumor aggressiveness. These data imply that T_reg_ and Th17 cells may concomitantly expand during tumor progression, with T_reg_ mainly suppressing protective effector T cell proliferation while sparing Th17 proangiogenic activities, fostering cooperatively tumor progression, and the metastatic process (46).

Nevertheless, recent data, in particular tumor systems, point out that T_reg_ may exert a protective role for the host (13, 47, 48). The connection between tumor and inflammation is a well-assessed process (49), but now it is clearly emerging that the type of tumor-associated inflammation imprints the behavior of T_reg_, becoming detrimental or beneficial for the host. Type-1 inflammation, characterized by high concentration of IFN-γ and IL-12 and fully active cytotoxic cells, represents efficient anti-tumor immunity (49). In this setting, the inhibitory properties of T_reg_ may promote tumor escape and aggressiveness (47). On the contrary, immune responses dominated by cytokines like TNF-α, IL-1β, IL-6, IL-23, and IL-17 act as pro-tumoral mediators (47). In this environment T_reg_ may suppress a pro-tumoral inflammatory status, thus playing a protective role for the host (47).

These unexpected anti-tumoral T_reg_ properties were observed in patients with colorectal cancer (CRC) (50–52). In these patients, with different tumor stages, a better prognosis and an increased overall survival were associated with higher infiltration of FOXP3^+^ T cells compared to patients with a poor tumor outcome. These data suggested the hypothesis that FOXP3^+^ T cells could be considered as an independent prognostic factor for CRC. Following a strong activation, both conventional CD4^+^ (53) and CD8^+^ (54) lymphocytes up-regulate Foxp3 expression in colonic tissue. These observations indicated that the CRC prognosis positively correlated with non-regulatory FOXP3^+^ cells rather than to T_reg_. However *in vitro* suppression assays demonstrated that FOXP3^+^ cells, isolated from CRC tissues, were endowed with suppressive functions, confirming their nature as regulatory cells (55). In a recent study conducted on 65 patients with different stages of CRC, FOXP3 expression was systematically evaluated in both tumor-infiltrating lymphocytes and neoplastic cells, and was correlated to tumor progression and clinical-pathological features (56). From this study the notion emerged that high FOXP3 expression in tumor cells correlated with poor tumor outcome, compared to tumors poorly expressing FOXP3; on the contrary, no correlation was observed between CRC prognosis and FOXP3 expression by T cells (56).

A protective role of T_reg_ was also found in head and neck squamous cell carcinoma (HNSCC) (57). Univariate and multivariate analysis demonstrated that the locoregional control of the tumor was positively associated with CD4^+^FOXP3^+^ regulatory cell infiltration (57). However, also for this type of neoplasia, there are some discordant data regarding the role of T_reg_ in tumor progression. Indeed, another study showed that T_reg_ frequency and suppressive function were higher in the peripheral blood of tumor-bearing patients than in healthy volunteers (58).

The discrepancies observed in these studies may be due to the number of patients included, different strategies of analysis and non-homogeneity of tumor samples (stage, metastasis, etiology). Certainly, to properly define the role of T_reg_ in tumor outcome, the new studies should take into account the tumor stage and the related inflammatory features, depending on the anatomical localization. In general, those tumors arising from chronic inflammation, almost at their initial stage, can benefit from the suppressive properties of T_reg_. In fact, during the inflammatory process, T_reg_ highly accumulate in the site of inflammation such to prevent exacerbated immune responses and tissue damage, which are the prelude to neoplastic transformation. On the contrary, in the presence of an established tumor, T_reg_ may reduce anti-tumor immunity thus favoring tumor escape. A more specific definition of T_reg_ contribution in tumor development and progression is desirable for the design of new and more effective immunotherapies, allowing the discrimination among tumors that will benefit or not from T_reg_ depletion/inhibition.

## Evidence for pT_reg_ or tT_reg_ Accumulation in Tumors

### Distinguishing features of pT_reg_ and tT_reg_

Many efforts have been recently addressed to the identification of phenotypic, molecular, and functional features distinguishing tT_reg_ and pT_reg_, besides their site of origin (11). Some markers have been proposed to distinguish pT_reg_ and tT_reg_, even though with some limitations: the initial enthusiasm for the suggestion of Helios as able to identify tT_reg_ (59) has been soon moldered by the observation of Helios expression in pT_reg_ (60); the recent finding of the Nrp-1 as a marker of tT_reg_ (61, 62) has application limited to murine cells, being not expressed on human T_reg_ (63). Several attempts have been conducted to identify genetic (64–66) and/or epigenetic signatures distinguishing pT_reg_ and tT_reg_. The T_reg_-specific demethylated region (TSDR) is involved in the stable commitment of the T_reg_ lineage, and controversy still remains on whether iT_reg_ or pT_reg_ can efficiently demethylate this region and become fully committed T_reg_ (66–69). Despite this growing amount of information, distinguishing the relative contribution of pT_reg_ and tT_reg_ to immune suppression in physiological and pathological conditions remains hard. However, some pieces of evidence have accumulated in the last years that speak in favor of tT_reg_ or pT_reg_ prevalence or concomitance in tumors.

### Evidence for tT_reg_ accumulation in cancer

One of the first attempts to distinguish between pT_reg_ conversion and tT_reg_ expansion in cancer was pursued by Bui and colleagues who adoptively transferred CD4^+^CD25^+^ cells, mixed at 1:10 ratio with CD25-depleted Thy1.1-congenic splenocytes, into immunodeficient mice bearing a progressive sarcoma (70). The analysis performed 10 days after tumor injection showed that the vast majority (around 80%) of tumor-infiltrating CD4^+^CD25^+^ cells derived from expansion/recruitment of the transferred T_reg_, rather than from conversion of non-T_reg_. This and other reports, appeared in the “pre-Foxp3” era, were biased by the idea that CD25 was the most stringent T_reg_ marker and that CD25-depleted cells represented a suitable precursor population to efficiently detect *de novo* generation of pT_reg_. However, subsequent studies have demonstrated that the CD25^+^ subset of Foxp3^−^ T_conv_ is enriched in pT_reg_ precursors (69, 71), thus the extent of pT_reg_ differentiation from CD25-depleted cells represents probably an underestimation of the actual contribution of pT_reg_ induction in the tumor context. Other authors have shown that tT_reg_ may dominate pT_reg_ not only quantitatively but also qualitatively, in terms of suppressive function: indeed, IDO^+^ plasmacytoid dendritic cells, derived from mouse tumor-draining lymph nodes, were capable to induce Foxp3^+^ pT_reg_ at very high levels but were unable to activate the suppressive function of these cells to an extent comparable to tT_reg_ (72). Many studies have clearly shown T_reg_ proliferation (in terms of *de novo* DNA synthesis and/or cell division) in tumor-bearing mice or cancer patients, thus indirectly supporting the idea that expansion of pre-existing tT_reg_ might prevail over pT_reg_ differentiation in building the tumor-associated T_reg_ pool. For instance, T_reg_ have been shown to incorporate high levels of BrdU in tumor-draining lymph nodes and at cancer sites in several experimental models (73, 74). A study conducted in patients with multiple myeloma showed that the TREC content in naive cells was significantly lower in T_reg_ (identified as CD4^+^CD25^high^ cells) than CD4^+^CD25^−^ or CD25^low^ cells, suggesting that the T_reg_ pool mainly derived from peripheral expansion rather than recent thymic emigration (75). However, the observation of high T_reg_ proliferation at tumor sites cannot be considered as an unequivocal proof of tT_reg_ prevalence over pT_reg_, since both subsets could be endowed with the same proliferative potential *in vivo*. Indeed, several pieces of evidence indicate that conversion and proliferation may represent uncoupled and independent events (see pTreg Development and tTreg Expansion as Independent Processes).

### Evidence for pT_reg_ induction in cancer

Some studies support the idea that pT_reg_ conversion actually occurs in tumor-bearing hosts at higher efficiency than in physiological conditions, even if controversy still exists on whether pT_reg_ may prevail numerically over tT_reg_ at the tumor site. We have in the past demonstrated that thymectomized and CD25-depleted mice, subsequently transplanted with carcinoma cells, showed a significantly higher T_reg_ recovery in tumor-draining than in contralateral lymph nodes, suggesting that in tumor-bearing mice the T_reg_ pool might be replenished mostly by newly derived pT_reg_ than by proliferation of residual T_reg_. To prove this possibility, CD25-depleted CD4-purified T cells were transferred into immunocompetent, Thy1.1-congenic, CT26 tumor-bearing mice. In this setting, we could show that the transferred cells acquired CD25 and Foxp3 at significantly higher levels in draining lymph nodes, compared to contralateral lymph nodes of tumor-bearing mice, or to the lymph nodes of tumor-free mice (76). This result clearly showed that tumor progression actively promoted the conversion of non-regulatory precursors into pT_reg_. Some tumor-derived molecular signals were found to be involved in tumor-associated conversion. For instance, in different mouse models, tumor cells have been shown to induce *in vitro* T_reg_ conversion through TGF-β, and TGF-β neutralization abrogated T_reg_ accumulation at the tumor site (77). Human leukemic cells converted *in vitro* non-T_reg_ into T_reg_ through the tumor cell-restricted IDO activity, and IDO blockade prevented pT_reg_ induction *in vivo* in a leukemia mouse model (78). A confirmation of extensive pT_reg_ infiltration in murine tumors has been recently obtained thanks to the recent discovery of Nrp-1 as a tT_reg_-restricted marker (61, 62). The analysis of Nrp-1 expression has indeed revealed that Nrp-1-negative *bona fide* pT_reg_ cells were significantly enriched at tumor site compared to spleen, ranging around 40–90% of total tumor-infiltrating T_reg_ depending on the tumor type (61). These Nrp-1-negative cells also presented a gene signature (Helios^low^, SWAP-70^low^, and Dapl1^high^) compatible with the pT_reg_ identity (61). Unfortunately, human T_reg_ do not express Nrp-1 (63), thus this marker cannot be used to estimate the relative contribution of tT_reg_ or pT_reg_ in human cancers.

## Developmental and Functional Relations between pT_reg_ and tT_reg_ in Cancer

### pT_reg_ development and tT_reg_ expansion as independent processes

Many attempts have been made to understand whether tT_reg_ accumulation and pT_reg_ development are mutually exclusive or rather cooperative in establishing immune suppression. The evidence that tT_reg_ may “educate” T_conv_ to convert into T_reg_ through the secretion of cytokines, such as TGF-β and IL-10 (79), may support the latter possibility. This event would generate a cascade of suppressive function transmitted from T_reg_ to bystander cells, establishing a loop of immunosuppression, reminiscent of a phenomenon called as “infectious tolerance” (80). Zhou and coworkers have addressed this issue in the tumor setting, and have demonstrated that tumor-antigen-specific pT_reg_ could indeed arise from T_reg_-depleted cells (adoptively transferred in mice carrying the cognate antigen-expressing tumor), but that the extent of pT_reg_ induction was not affected by the concomitant presence of tT_reg_, either exogenous (adoptively co-transferred) or endogenous (pre-existing in the host) (81, 82). This result indicated that tT_reg_ and pT_reg_ accumulate in tumors in a reciprocally independent fashion and that “infectious tolerance” may play minor roles in shaping the tumor-associated T_reg_ pool.

A comprehensive scenario of T_reg_ accumulation in tumors should take into account, beside *de novo* conversion, the active proliferation of not only tT_reg_ but also pT_reg_. Proliferation plays opposite roles in the differentiation of T_conv_ into pT_reg_ *versus* the expansion of already differentiated pT_reg_. Regarding the former aspect, we have demonstrated that T_conv_ proliferation was not required for their conversion into pT_reg_, since CD25^+^Foxp3^+^ cells could develop in tumor-bearing mice from CD25-depleted cells treated with an anti-proliferative agent (76). A seminal study by Kretschmer and colleagues showed that T_conv_ proliferation was not only dispensable but also detrimental to conversion: indeed, low levels of T cell proliferation, in conditions of suboptimal antigen presentation, lack of co-stimulation, and IL-2 paucity, favored TGF-β-mediated pT_reg_ induction, thus suggesting that an inverse relationship might exist between T_conv_ proliferation and conversion into pT_reg_ (83). However, once developed, pT_reg_ promptly proliferated in response to experimental antigens (83) and, more importantly, in response to tumor antigens (81, 82). Experiments performed in CNS1-mutated mice, which are genetically unable to generate pT_reg_, have shown that pT_reg_ and tT_reg_ may occupy distinct “niches”: indeed, the efficiency of pT_reg_ differentiation from T_conv_ was higher when those T_conv_ were co-transferred, in lymphopenic recipients, with a CNS1-deficient (non-containing pT_reg_) compared to a CNS1-sufficient (containing pT_reg_) counterpart, suggesting that not only the tT_reg_ pool, but also the pT_reg_ niche, may be controlled by autonomous homeostatic mechanisms (84).

### Division of labor between tT_reg_ and pT_reg_ in cancer

Both tT_reg_ and pT_reg_ have been generally recognized as immune suppressive cells in a variety of *in vivo* and *in vitro* experimental settings (12). However, whether the two subsets are endowed with peculiar activities remains unclear and is a matter of intense investigation.

Gene expression profiling revealed that the pT_reg_ and tT_reg_ signatures were mostly overlapping but also presented some differentially expressed genes, encoding for proteins involved in T_reg_ suppressive function, suggesting that pT_reg_ may preferentially exploit different molecules and related mechanisms to exert suppression (64–66). The Nrp-1 itself is not only a marker discriminating murine tT_reg_ from pT_reg_, but also plays a role in T_reg_ suppression: since this molecule prolongs T_reg_ interactions with immature dendritic cells, tT_reg_ may benefit from this pathway in preferentially modulating dendritic cell and cognate T cell activation (22). Many data suggest that pT_reg_ may be specialized suppressors of immune responses at interfaces with external environments, such as airways, gut, and maternal-fetal interface (64, 84–87). Of note, a peculiar T_reg_ suppressive molecule, IL-10, plays crucial roles at environmental interfaces, therefore pT_reg_ may perform their specialized activity through IL-10 secretion (34, 88). IL-10 is critically involved in the establishment of tumor-associated immune suppression, and we have clearly demonstrated IL-10 production by around 40% of tumor-infiltrating T_reg_ in murine transplanted tumors (36). It would be interesting to understand whether the fraction of IL-10-producing T_reg_ is enriched in pT_reg_, rather than tT_reg_, in tumors. One study has directly addressed the issue of induced T_reg_ functional specialization in tumors, by generating *in vitro* tumor-specific iT_reg_ and co-culturing these cells, or tT_reg_ as control, with NK cells: these authors found that iT_reg_ and tT_reg_ equally suppressed IL-2-induced NK activation, but only iT_reg_ were endowed with the surprising ability not to suppress, but to enhance, NK cytotoxicity induced by tumor target cell contact (89). This observation may speak in favor of differential roles played by tT_reg_ and pT_reg_ in cancer, with the former more involved in preventing target cell-independent, and possibly self-directed, unwanted immune responses, and the latter concurrently enhancing tumor-specific immunity.

This division of labor may result in the progressive shaping of the immune response toward an effective anti-tumor immunity with minimal side effects. Such dichotomy is also reminiscent of the double role played by T_reg_ in different cancers, according to the hypothesis that high T_reg_ frequency is associated to poor or good prognosis in non-inflammatory or inflammatory cancer onset, respectively (13, 47). In the former case, i.e., non-inflammatory cancers in which protective type-1 responses are suppressed by high T_reg_ infiltration, T_reg_ may mainly recognize tumor-associated self-antigens, and mostly include tT_reg_; conversely, in the case of inflammatory cancers, related to chronic low-dose type-17 cytokines, which are typical of mucosal tissues, high numbers of pT_reg_ may suppress pro-tumoral inflammation through IL-10, relevantly produced by pT_reg_ at those sites. We are tempting to speculate that tT_reg_ may dominate in suppressing anti-tumor type-1 responses, while pT_reg_ may prevail in shaping pro-tumor type-2 and type-17 inflammatory responses. Notably, the prototypical example of an inflammation-related tumor in which T_reg_ accumulation associates to good prognosis is CRC (50), developing in the gastrointestinal mucosa, in which immune tolerance is under the control of pT_reg_ (84).

### Antigen specificity of tT_reg_ versus pT_reg_ in cancer

Antigen recognition may play a crucial role in dictating whether tT_reg_ or pT_reg_ will prevail in the tumor context. Controversy still exists on the antigen specificity of these two populations. On the one side, tT_reg_ are generally believed to recognize self-antigens encountered during thymic selection (90). On the other side, pT_reg_, deriving from T_conv_, are thought to display the same TCR repertoire of T_conv_ and thus to mainly recognize foreign antigens. Indeed, only a small overlap exists between TCR repertoires of pT_reg_ and tT_reg_ (66), and pT_reg_ are believed to recognize non-self-antigens such as commensal microbiota, allergens, and fetal alloantigens (84, 87).

Tumor cells can express a variety of antigens that can be broadly classified into: (i) self-antigens physiologically expressed as in the tissue of origin, (ii) self-antigens aberrantly expressed, in terms of expression level, developmental stage, or histotype (called tumor-associated antigens or TAAs), and (iii) neo-antigens uniquely expressed by tumor cells, mostly derived from oncogenic mutations (named tumor-specific antigens or TSAs). Based on the above considerations, self-antigens and TAA should be recognized by tT_reg_, while TSA would induce and activate pT_reg_. However, a complex picture arises from studies analyzing the TCR specificity of tumor-associated T_reg_.

#### T_reg_ can recognize TAA and TSA in tumors

In different tumor models, TCR-transgenic T_reg_ have been shown to promptly proliferate in response to the cognate antigen specifically expressed by tumor cells, suggesting that T_reg_ can undergo tumor-antigen-driven activation and expansion (74, 81, 82, 91). Antigen presentation in the tumor context may favor T_reg_ expansion: in a mouse model of spontaneous prostate cancer, an efficient T_reg_ induction/expansion occurred only when TCR-transgenic, antigen-specific CD4 T cells encountered the cognate antigen expressed in the context of prostate cancer cells, rather than non-transformed cells or viral vector-infected cells (91). In this model, TAA-specific T_reg_ were recognized as pT_reg_ induced *in vivo* in a TGF-β-independent fashion.

This evidence of TAA-responding T_reg_ has been confirmed in human tumors. CD4 clones derived from cancer patients resulted to be regulatory cells and to recognize peptides derived from TAAs, such as LAGE1 (92), ARTC1 (93), TRAG-3, NY-ESO-1 (94–96), Melan-A (97), survivin, TRP1, and gp100 (94) in melanoma patients, and WT-1 in leukemia patients (98). By using MHCII/peptide tetramer technology, other authors failed to detect T_reg_ specific for NY-ESO-1 in the peripheral blood of ovarian cancer patients (99). Bonertz et al. developed an *in vitro* system to screen the suppressive function of T_reg_ in response to single peptides and, with this approach, could detect T_reg_ specific for several TAA in the peripheral blood of colon carcinoma patients but not in healthy donors; notably, T_reg_ depletion *in vitro* unveiled TAA-specific T_conv_ responses (100).

The possibility that tumor-associated T_reg_ may recognize not only TAA but also TSA is demonstrated by the observation that T_reg_ specific for exogenous viral antigens, acting as TSA, may arise in virus-related cancers. T_reg_ clones specific for human papilloma virus (HPV), and suppressing the cognate antigen-directed T cell response, have been obtained from tumor-draining lymph nodes and tumor biopsies of cervical cancer patients (101). T_reg_ clones specific for antigens of the Epstein–Barr virus (EBV), associated to several hematological and solid malignancies, can be generated from the peripheral blood of healthy donors (102).

#### T_reg_ can recognize self-antigens in tumors

Several data in mouse models confirm that T_reg_ responding to self-antigens can play a role in suppressing the anti-tumor responses. Immunization with serologically defined auto-antigens was found to enhance tumor growth in different mouse models, and this event was dependent on the expansion of T_reg_ responding to those self-antigens (103). This study confirmed that self-antigens-specific T_reg_ could suppress anti-tumor immunity, but did not explore the T_reg_ response to self-antigens expressed by tumor cells themselves during tumor progression. This issue has been instead addressed in an experimental model in which a foreign antigen, artificially expressed in transgenic mice under tissue-specific promoter, was seen (peripherally and/or thymically) by the immune system as a self-antigen and elicited the generation of a pool of memory T_reg_ specific for that antigen (74). If those mice were injected with the cognate antigen-bearing tumor, the memory T_reg_ pool specific for that self-antigen was hugely expanded in tumors and tumor-draining lymph nodes, confirming that self-specific T_reg_ can respond to self-antigens expressed by tumor cells (74). A seminal paper has recently reported the immunoscope analysis of T_reg_ infiltrating spontaneous prostate tumors in a mouse transgenic model, and described the clonal enrichment of a single T_reg_ specificity that was directed not to a unique TSA but to a self-antigen expressed also by normal prostate cells (104). The development of T_reg_ specific for peripheral tissue-restricted self-antigens occurred in the thymus under the control of the Aire molecule, which allows the expression of peripheral antigens in thymic epithelial cells (104). These findings clearly demonstrate that T_reg_ can recognize self-antigens in cancer and suggest that maintaining self-antigen expression may help transformed cells to overcome the immune surveillance through self-specific T_reg_ expansion.

#### Repertoire analysis as an estimation of pT_reg_/tT_reg_ balance

The direct comparison between the repertoires of tumor-associated T_reg_ and T_conv_ may help understanding the processes underlying T_reg_ enrichment in cancer. Some authors have reported that the analysis of TCR diversity (performed with the immunoscope technology) showed that T_reg_ infiltrating murine transplanted tumors displayed a biased TCR repertoire toward “public” CDR3 sequences (i.e., shared by different mice), suggesting T_reg_ intra-tumor clonal expansion driven by the recognition of dominant antigens (105). Also tumor-infiltrating activated T_conv_ showed a biased TCR repertoire, but it was distinct from the T_reg_ spectrum, and the minimal overlap between the two subsets was mainly confined to “private” specificities (105). By using a similar approach, others have reported distinct and not overlapping TCR repertoires of T_reg_ and T_conv_ infiltrating prostate tumors in a genetically engineered mouse model of spontaneous prostate carcinogenesis (104). Lack of overlap between T_conv_ and T_reg_ repertoires was also found in tumors and tumor-draining lymph nodes in a mouse model of chemical carcinogenesis (106). Overall, the lack of overlap between T_reg_ and T_conv_ has been interpreted in many cases as the result of negligible pT_reg_ conversion at the tumor site; however, pT_reg_ and tT_reg_ may share more specificities than expected, since tT_reg_-associated antigens may preferentially drive T_conv_ fate decision toward the conversion into pT_reg_ rather than toward the conventional activation; moreover, already established pT_reg_ may then undergo intra-tumor clonal expansion together with tT_reg_ in response to the same antigens. Therefore, the overall overlap between T_conv_ and T_reg_ specificities may not accurately estimate the extent of pT_reg_ induction in tumors. Indeed, in one study performed in advanced melanoma patients, TAA-specific TCRs, expressed by tumor T_reg_ clones, could be detected in both T_reg_ and T_conv_ populations, demonstrating that TAA-specific T_reg_ may be comprised of pT_reg_ derived from the conversion of T_conv_ (95).

Indirect data support the notion that TAA-specific T_reg_ may contain pT_reg_. TAA-specific T_reg_ clones, obtained from patients with advanced melanoma, suppressed *in vitro* the cognate antigen-specific T cell response, but produced high levels of Th1 and/or Th2 cytokines (95), and showed low FOXP3 expression and TSDR demethylation, indicating that these cells may represent an incompletely uncommitted T_reg_ population, which more likely belongs to the pT_reg_ than to the tT_reg_ pool (95).

A recent study has directly evaluated the consequences of pT_reg_ and tT_reg_ antigen specificities in tumor-bearing hosts. Indeed, Schreiber et al. have shown that, if purified polyclonal tT_reg_ and T_conv_, differentially labeled with green or red fluorescence, were co-transferred in CD4-null mice, the tT_reg_ progeny exceeded the newly T_conv_-derived pT_reg_ population in tumor-draining lymph nodes as well as in the spleen; conversely, when transgenic, tumor-antigen-specific, tT_reg_ and T_conv_ were injected, tT_reg_ and pT_reg_ reached comparable frequencies in tumor-draining lymph nodes (107). These results suggest that tT_reg_ and pT_reg_ are mostly specific for self- or tumor-antigens respectively, and that the balance between pT_reg_ and tT_reg_ may be fine-tuned by the relative prevalence of TSAs versus self-antigens expressed by tumor cells.

## Heterogeneity and Plasticity of tT_reg_ and pT_reg_

### T_reg_ heterogeneity in cancer: Relations with the pT_reg_/tT_reg_ dichotomy

**Figure 2:**
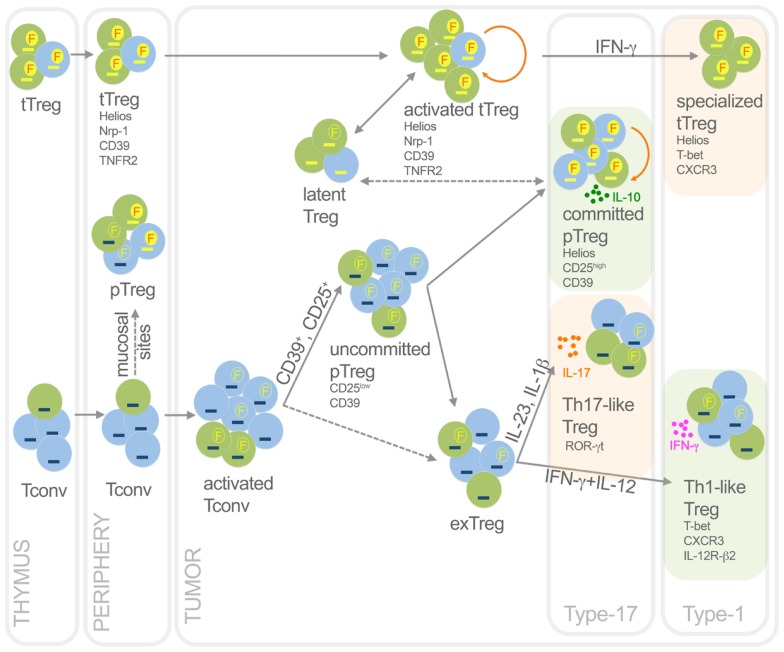
**Functional dynamics of tT_reg_ and pT_reg_ in cancer**. This picture summarizes development, heterogeneity, plasticity, antigen specificity, and function of pT_reg_ and tT_reg_ in cancer. Activated T_reg_, which are epigenetically committed and mostly self- and TAA-specific, can transiently lose Foxp3 without methylating TSDR thus becoming latent T_reg_; in some conditions, they can acquire T-bet expression thus becoming specialized suppressors, detrimental to the anti-tumor type-1 response. Activated T_conv_, mostly foreign (TSA) antigen-specific, can promiscuously express Foxp3 without demethylating TSDR. However, a fraction (CD25^+^, or CD39^+^) of activated T_conv_ can convert into pT_reg_, progressively moving from an uncommitted to a committed stage. Through IL-10, committed pT_reg_ can suppress pro-tumoral inflammatory and type-17 responses, thus exerting beneficial roles for the host in some cancer types. In some contexts, uncommitted pT_reg_ (and possibly activated T_conv_) can move back to exT_reg_ stage, acquiring the ability to produce inflammatory cytokines. Therefore, in some tumors such as colon cancer, Th17-like T_reg_ may foster type-17 inflammation thus supporting tumor growth; in other tumor contexts, Th1-like T_reg_ can favor type-1 responses that rather block tumor growth. Green, cells specific for self-antigens and TAA; light blue, cells specific for foreign antigens including TSA. Yellow dash, demethylated TSDR; blue dash, methylated TSDR. Red “F” in yellow circles, stable Foxp3; yellow “F” in empty circles, unstable Foxp3. Dashed arrows, unclear events. Orange rounded arrows, proliferation in the tT_reg_ or the pT_reg_ homeostatic niche. Light green frames, conditions in which T_reg_ are beneficial to the host; light orange frames, conditions in which T_reg_ are detrimental to the host.

During the latest years, it has become increasingly clear that T_reg_, meant as Foxp3-positive cells, are not a homogeneous lineage, but rather represent a mixture of subpopulations. Indeed, beside the tT_reg_/pT_reg_ distinction based on their developmental origin, diverse T_reg_ subsets can be identified endowed with peculiar features in terms of suppression, proliferation, and stability, even though not properly classifiable as developmentally distinct lineages (Figure 2). Tumor microenvironmental signals may differentially affect these subsets, thus shaping T_reg_ heterogeneity to the advantage of tumor progression.

#### T_reg_ stability and epigenetic commitment in cancer

Foxp3 inherent stability, rather than Foxp3 expression in a given moment and tissue, is intimately linked to an actual commitment to the T_reg_ lineage and therefore to the maintenance of immune suppression. Pioneer studies have demonstrated that Foxp3 stability is strictly related to an epigenetic imprinting of CpG demethylation in the TSDR region of the *Foxp3* locus (67, 86, 108). TSDR demethylation was then recognized as the mechanism featuring the distinction between committed (demethylated) and uncommitted (methylated) T_reg_, irrespective of Foxp3 expression (9). Controversy exists on whether pT_reg_ show complete or partial TSDR demethylation and can then be considered as committed T_reg_. Many studies show that iT_reg_ have a partially or completely methylated TSDR (9, 67–69), while pT_reg_ have been described as TSDR-demethylated (68), TSDR-methylated (66), or as a mixed population of stable and unstable cells, characterized by CD25 high or low expression respectively (69).

Few data exist on the extent of TSDR demethylation in tumor-associated T_reg_. The frequency of TSDR-demethylated cells is higher in peripheral and intratumoral leukocytes of lung, colon, prostate, or breast cancer patients, in relation to a higher T_reg_ frequency as determined by flow cytometry or immunohistochemistry (109). Of note, the extent of TSDR demethylation in CRC patients was only slightly higher than in healthy volunteers, in contrast to the significantly increased T_reg_ frequency in these samples shown by previous studies (110, 111). This discrepancy may be explained with the peculiar nature of this inflammation-related and mucosal tissue-located cancer, in which the inflammatory response may specifically involve pT_reg_, possibly containing more uncommitted (TSDR-methylated) cells than tT_reg_.

The evaluation of TSDR demethylation has been used as a reliable analytical tool for the estimation of committed T_reg_ in some tumor conditions and therapies. An increased frequency of epigenetically committed (TSDR-demethylated) T_reg_ has been determined in tumor-infiltrating cells of ovarian, colorectal, and bronchial cancers compared to non-tumoral tissue counterparts (112). TSDR demethylation was decreased in the peripheral blood of metastatic renal carcinoma patients receiving tumor vaccination (113), and increased in patients treated with dendritic cell vaccination and cytokine therapy (114).

#### T_reg_ functional dynamics in cancer

The idea of Foxp3 as the master transcription factor of T_reg_ lineage has been challenged by the observation that some T_reg_ features are Foxp3-independent, and that Foxp3 plays T_reg_-unrelated functions (115). This is especially true for human FOXP3-positive cells, since human activated T_conv_ can transiently express this transcription factor that acts as an intrinsic T cell regulator (116). The concomitant analysis of CD45RA and FOXP3 in human T_reg_ in both physiological and pathological contexts allowed delineating a classification into three subsets: CD45RA^+^FOXP3^low^ resting T_reg_ (rT_reg_), CD45RA^−^FOXP3^low^ non-T_reg_, and CD45RA^−^FOXP3^high^ (CD45RO^+^) activated T_reg_ (aT_reg_), endowed with different potentials of proliferation, suppression, stability, and plasticity (117). Whether each subset mainly contains tT_reg_ or pT_reg_ is unclear. While rT_reg_ were recognized as CD31^+^ recent thymic emigrants, thus belonging to the tT_reg_ pool, aT_reg_ can be considered as a mixed population of activated tT_reg_ (derived from rT_reg_) and pT_reg_ (derived from non-T_reg_ or T_conv_). The CD45RA^−^FOXP3^low^ non-T_reg_ subset may represent a mixture of activated T_conv_ (promiscuously and unstably expressing FOXP3), latent T_reg_ (transiently downregulating FOXP3), and recently converted pT_reg_ (117).

The three human T_reg_ subsets can be differentially expanded in distinct pathologies. In conditions characterized by exacerbated immune responses, such as autoimmune diseases, rT_reg_ and non-T_reg_ are expanded; conversely, in diseases associated to immune unresponsiveness, such as sarcoidosis, the aT_reg_ subset is instead enriched in the peripheral blood (117). The tumor context, which conceivably belongs to the latter category, should be characterized by aT_reg_ expansion. In line with this hypothesis, CD45RO^+^FOXP3^high^ aT_reg_ were found significantly expanded in the peripheral blood, and much more at the tumor site, in patients with malignant melanoma (118). Also the non-T_reg_ and the rT_reg_ fractions were increased, but only in the peripheral blood, in cancer patients compared to healthy controls, and both subsets positively correlated with tumor progression (118). The non-T_reg_ pool produced some IFN-γ and its frequency returned to normal levels after tumor removal, thus probably representing aberrantly activated T_conv_, or T_reg_ with attenuated FOXP3 activity (118). A much deeper knowledge on T_reg_ dynamics in cancer is needed to better understand the role played by each specialized component in suppressing anti-tumor type-1, or pro-tumor inflammatory, responses.

#### T_reg_ subsets specified by functional/developmental markers

Several surface or intracellular markers have been suggested to identify T_reg_ subsets endowed with peculiar abilities other than suppressive functions. A portion of human T_reg_ with an effector/memory-like phenotype (26, 119, 120) expresses CD39, which has been proposed as a target to enrich human suppressive T_reg_ (119). CD39 was found overrepresented in peripheral and tumor-infiltrating T_reg_ from HNSCC, and was further increased in patients with advanced-stage disease or after radiochemotherapy (120, 121). CD39 is also expressed by a T_conv_ subset, which produces lower levels of pro-inflammatory cytokines than the bulk T_conv_ population, and is more prone to convert, at least *in vitro*, into T_reg_ (120). Both CD39^+^ T_reg_ and CD39^+^ T_conv_ were enriched in peripheral blood, and further increased at tumor site, in HNSCC patients, and a positive correlation existed between frequencies of these two populations (120). Therefore, these data suggest that tumor-associated CD39^+^ T_conv_ may represent a reservoir of CD39^+^ T_reg_ precursors. As a consequence, it could be suggested that CD39^+^ T_reg_ may include both tT_reg_ and pT_reg_, and that both T_reg_ subsets can exploit the CD39-mediated suppressive mechanisms of ATP degradation and adenosine generation.

Not only the functional arms of suppression, but also the activation requirements may differ in tT_reg_ and pT_reg_: for instance, TNFR2 expression is needed to activate tT_reg_ but not iT_reg_ suppressive ability in experimental colitis (122). Of note, TNFR2-positive T_reg_ have been found enriched in murine tumors, in association with a higher suppressive ability, *ex vivo*, in the standard suppression assay (123). In a mouse model of metastatic melanoma, TNF-α caused enhanced tumor progression through the TNFR2-mediated T_reg_ expansion at the site of metastasis (124). These data suggest that TNFR2 expression may label tumor-infiltrating T_reg_ of thymic origin, and that TNF-α at the tumors site may preferentially expand and activate tT_reg_. Supporting the idea that tT_reg_ may represent more stable cells, TNFR2 was found to be involved in the maintenance of Foxp3 stability in mouse models of inflammation (125). Also in human peripheral blood, CD25 and TNFR2 co-expression identifies cells highly expressing FOXP3, showing an effector/memory phenotype and strong suppression, *ex vivo* (126). The TNF-α/TNFR2 pathway may amplify T_reg_ activation also through the induction of a NF-kB-driven transcriptional program enriched for other members of TNF superfamily, such as 4-1BB, FAS, and OX40 (127).

The early idea that Helios could differentiate tT_reg_ from pT_reg_ (59, 128) prompted the use of this marker in delineating tT_reg_ accumulation in cancer. The vast majority of tumor-infiltrating T_reg_ were found to express Helios in a mouse model of glioblastoma (129), in glioblastoma multiforme patients (129), and renal cell carcinoma patients (130). However, the value of Helios as univocal marker of tT_reg_ has been questioned by other studies that showed Helios also expressed in pT_reg_ (60, 131), and in association to T_reg_ suppression (128, 131) and commitment. Indeed, Helios^−^FOXP3^+^ cells freshly isolated from healthy donors or autoimmune disease patients showed decreased TSDR demethylation compared to Helios^+^FOXP3^+^ (132, 133), and also displayed a higher plasticity in terms of cytokine production (133). In a murine transplanted tumor model, tumor-infiltrating T_reg_ were enriched in Helios^+^ cells, representing the subset with the highest proliferative potential (as shown by Ki67 staining) (131). In summary, the well-recognized enrichment of Helios^+^ T_reg_ in several human and mouse tumors may be attributed, rather than to preferential attraction and expansion of tT_reg_, to the tumor-driven local activation and/or commitment of both tT_reg_ and pT_reg_.

### Specialization and plasticity of tT_reg_/pT_reg_ in cancer

It is now well established that T_reg_ (or better, their specific subsets) adapt their molecular programs to optimize their *in vivo* suppressive function in distinct inflammatory milieus, which may be alternatively dominated by Th1, Th2, Th17, or T_FH_ responses. Strikingly, these T_reg_ specialized programs are orchestrated by the same transcription factors that drive the polarization of the targeted T-helper subset: therefore, T-bet, IRF4, Stat3, and Bcl6 expression are respectively and selectively required for the T_reg_ specialized suppression of Th1 (134, 135), Th2 (136), Th17 (137), and T_FH_ (138, 139) responses. Indeed, by acquiring master T-helper genes, T_reg_ may gain the expression of chemokine receptors driving the recruitment of specialized T_reg_ into inflamed tissues. However, in some contexts, T_reg_ (or, again, some T_reg_ subsets) can express not only T-helper-related transcription factors and migratory molecules, but also cytokines such as IFN-γ or IL-17, thus turning from specialized suppressors into so-called Th1-like or Th17-like T_reg_ that may rather contribute to inflammation (140). Some data, mostly from mouse experimental models, suggest that such T_reg_ plasticity is not a lineage reprograming of committed T_reg_, which appear instead quite stable; rather, Th1-like or Th17-like T_reg_ may derive from uncommitted cells expanded in inflammatory conditions (69, 141). However, other studies have shown that in both mouse and human pathologies T_reg_ can produce relevant amounts of type-1 and type-17 cytokines even though preserving Foxp3 expression (142–146).

#### Th17-like T_reg_ in cancer

Regulatory T cells may shift to a Th17-like phenotype in inflamed microenvironments dominated by type-17 cytokines, thus favoring, rather than suppressing, pro-tumoral mechanisms of chronic inflammation. According to this idea, human T_reg_ have been found to spontaneously secrete IL-17 in the intestine of patients carrying inflammatory bowel disease (145, 147) and colon carcinoma (147). In epithelial ovarian cancer, a malignancy associated to chronic inflammation, T_conv_ were found to secrete high levels of IL-17 (and other cytokines) when cultured *ex vivo* with IL-2; under similar conditions, tumor-infiltrating T_reg_ were prone to FOXP3 downregulation, attenuation of suppressive function, and prompt IL-17 production (148). In human lung adenocarcinoma, FOXP3 message amounts correlated with Th17-related transcripts enriched at the tumor site, where IL-17 antagonized the development of the anti-tumor, T-bet-dependent, Th1 response (149). Myeloid antigen-presenting cells and cytokines such as IL-2, TGF-β, IL-1, IL-23, and IL-6 may initiate T_reg_ polarization into Th17-like cells in these tumor contexts (147–149). In a mouse model of hereditary colon polyposis, as well as in human colon cancer, the Th17-like T_reg_ co-expressed the Th17-related transcription factor ROR-γt, and fostered tumor progression, also through the promotion of mast cell local expansion (150, 151). This study clearly demonstrated that microenvironmental signals could direct T_reg_ plasticity toward pro-inflammatory and pro-tumoral activities.

One group has demonstrated that Th17-like T_reg_ can also arise in experimental tumors as an outcome of vaccination strategies (152). In this study, vaccination with antigen plus TLR-9 ligand induced T_reg_ reprograming into polyfunctional T-helper-like cells, producing a wide array of cytokines including IL-2, TNF-α, and IL-17, and expressing cell-surface CD40L, thus providing efficient T cell help for tumor-antigen cross-presentation and development of anti-tumor response (152). The IDO immunosuppressive enzymatic activity was responsible for preventing this anti-tumor T_reg_ polarization, which was instead enhanced using an IDO blocker (152).

Little data exist on the precursors of Th17-like T_reg_ in cancer. In the peripheral blood of healthy subjects, the CD45RA^−^FOXP3^low^ non-T_reg_ subset was found enriched in Th17-related transcripts and in cells actively secreting IL-17, even at higher levels than naïve or memory T_conv_, a data suggesting that this population contains Th17 or Th17-like precursors (117). It would be interesting to understand whether the Th17 potential resides, within the non-T_reg_ gate, in activated T_conv_, in latent T_reg_, and/or in recently induced pT_reg_, possibly co-expressing FOXP3 and RORγt and thus pre-committed to the Th17 lineage.

#### Th1-like T_reg_ in cancer

Pioneer studies from Koch and colleagues demonstrated that, following exposure to IFN-γ in Th1-dominated microenvironments, a subset of T_reg_ can up-regulate the Th1-related transcription factor T-bet, which drives T_reg_ expansion, migration (CXCR3-mediated), and function specifically during type-1 inflammation (134). Further experiments have shed light on the developmental requirements and the alternative fates of murine T-bet^+^ T_reg_: following IFN-γ stimulation, T_reg_ could gain T-bet expression but failed to fully polarize into IFN-γ-producing Th1-like T_reg_, due to an impaired T_reg_ susceptibility to IL-12. Indeed, IL-12 receptor β2, which is inducible in T_conv_ in an IFN-γ- and T-bet-dependent fashion, is epigenetically inaccessible in T_reg_ (135). Only long-lasting IFN-γ preconditioning could unlock IL-12 responsiveness, thus allowing the complete T_reg_ polarization into Th1-like cells (135). Presumably, in contexts characterized by chronic IFN-γ and IL-12 abundance, such as autoimmune, inflammatory, and viral diseases, T_reg_ will be oriented to a full reprograming into Th1-like cells. Supporting this idea, IFN-γ-producing T_reg_ have been reported in mouse models of graft-versus-host disease (153), viral (154) or parasite (142) infection, in human multiple sclerosis (144), and diabetes (146, 155). In one of these systems, IFN-γ-producing T_reg_ were recognized to be specific for a foreign (viral) antigen (154). Whether such Th1-like T_reg_ can be yet considered as classical regulatory cells is still debated. One study has shown that *in vitro* polarized Th1-like T_reg_ were less suppressive than conventional T_reg_ in the standard *in vitro* suppression assay, and that suppression could be partially rescued with concomitant IL-10 and IFN-γ neutralization (144). Another study has proven that IFN-γ-producing human iT_reg_ were equally functional as natural T_reg_ in suppressing both proliferation and cytokine production of responder T cells (156). In a mouse model of graft-versus-host disease, IFN-γ produced by stable (TSDR-demethylated) T_reg_ was shown to be even required for T_reg_ protective effect (153), suggesting that IFN-γ-releasing T_reg_ can display *in vivo* unexpected functions depending on the context.

Conversely, it could be envisaged that, in the tumor context, the low levels of IFN-γ derived from T_conv_, NK, and CD8 cells, and the paucity of IL-12 production by tumor-associated APCs, may concur to induce a pool of T_reg_ expressing T-bet but not secreting IFN-γ, thus specialized in suppressing anti-tumor type-1 immunity. In line with this possibility, TAA-specific T_conv_, but not TAA-specific T_reg_, produced IFN-γ in patients with epithelial ovarian cancer (99). In both healthy subjects (117) and malignant melanoma patients (118), IFN-γ-producing cells were enriched within the circulating CD45RA^−^FOXP3^low^ (CD45RO^+^) non-T_reg_ subset, mostly including activated T_conv_ and/or uncommitted T_reg_. Conversely, in ovarian cancer, tumor-infiltrating T_reg_ were enriched in CXCR3^+^ cells, highly expressing T-bet but poorly producing IFN-γ, and strongly suppressing Th1 response *ex vivo* (157). Tumor-associated CXCR3^+^ T_reg_ were mostly Helios-positive, and T-bet^+^ T_reg_ could be generated *in vitro* by culturing CD45RA^+^CCR7^+^ rT_reg_ (mostly containing tT_reg_) under Th1-polarizing conditions (157), suggesting their derivation from committed tT_reg_. This finding was in accordance to Koch et al. who showed that T-bet^+^ T_reg_ derived from T-bet^−^ T_reg_, rather than from activated T_conv_ (134). These data support the idea that tT_reg_, rather than pT_reg_, may contain the precursors for Th1-specialized suppressors, thus playing critical roles in suppressing protective responses in tumors whose high T_reg_ frequency correlates with poor prognosis (13).

Some therapeutic interventions can force tumor-associated T_reg_ toward a fully differentiated Th1-like phenotype. For instance, circulating T_reg_ from melanoma patients showed significantly higher IFN-γ secretion following a protocol of tumor peptide vaccination plus IL-2 and cyclophosphamide, in line with enhanced serum IL-12 (158). On the whole, these data suggest that, especially in the human system, the transition from T-bet^+^ T_reg_, specialized Th1 suppressors, into T-bet^+^ IFN-γ^+^ T_reg_, Th1-like plastic cells, may not only depend on the availability and the responsiveness to exogenous stimuli, but may differentially occur in distinct T_reg_ precursors: on the one side, tT_reg_, enriched in committed and self-specific cells, may be forced to arrest to the specialization (T-bet^+^) endpoint; on the other side, pT_reg_, containing less committed and foreign antigens-specific cells, may be more prone to the complete reprograming into pro-inflammatory (T-bet^+^ IFN-γ^+^) cells. Future studies will elucidate the mechanisms by which different growing tumors may favor the expansion of pro-tumoral specialized Th1 suppressors or the induction of Th1-like plastic T_reg_.

## Implications for Cancer Immunotherapy

The initial enthusiasm on the use of therapeutic cancer vaccines has been soon disappointed by the observation of a low response rate in many trials (159). After the discovery of T_reg_ as potent immune suppressive cells hampering the establishment of anti-tumor immunity, it soon became clear that anti-tumor vaccination might fail to elicit an effective immune response and to achieve successful tumor eradication, because of the immune suppressive barrier created by T_reg_. In addition, since T_reg_ may recognize TAA and TSA at higher frequency than T_conv_, tumor-antigen-based vaccines may expand/induce T_reg_ rather than effector cells, thus inhibiting rather than boosting the anti-tumor response. Indeed, Zhou et al. first demonstrated that TCR-transgenic CD4 T cells specific for a TAA, adoptively transferred into mice bearing TAA-expressing tumor cells, proliferated extensively after administration of a therapeutic tumor vaccine (in the form of a recombinant vaccinia virus encoding the antigen), but tumor-antigen-experienced cells were mostly regulatory cells, *ex vivo* suppressive, and anergic to subsequent stimulation (81).

In cancer patients receiving tumor-antigen vaccination, the expansion of antigen-specific T_reg_ has been documented. Circulating NY-ESO-1-specific T_reg_ spontaneously develop in late-stage melanoma patients and are expanded following immunization with NY-ESO-1 protein supplemented with adjuvants (96). Therapeutic vaccination with an HPV synthetic long peptide vaccine, administered to patients with HPV-positive cervical carcinoma, induced both CD8 and CD4 T cell immunity, but also enhanced the HPV-specific T_reg_ pool (160). The pool of vaccine-specific T_reg_ may derive not only from the expansion of pre-existing tumor-antigen-specific clones, but also from *de novo* generation of vaccine-specific pT_reg_. This is suggested by results obtained vaccinating melanoma patients with an HLA-DP4-restricted MAGE-A3 peptide: in this setting, a subset of vaccine-specific T_reg_ becomes detectable only after vaccination (161). Vaccine-elicited T_reg_ showed some degree of heterogeneity: out of five CD25^+^ regulatory clones isolated from vaccinated patients, four expressed high FOXP3 mRNA levels, produced TGF-β, and showed demethylated TSDR; one clone expressed less FOXP3, had methylated TSDR and produced some Th2 cytokines (161). These data suggest that antigen-specific T_reg_, induced in the periphery following antigen exposure and thus recognizable as pT_reg_, can contain both committed and uncommitted cells.

The concomitant and detrimental T_reg_ expansion in anti-tumor vaccination can be avoided by using CD8 T cell-targeted approaches. A melanoma vaccination protocol based on an MHCI-restricted Melan-A peptide significantly decreased the frequency of Melan-A-specific T_reg_, in association with an improved and more diverse Th1 response (97).

Some attempts have also been made to combine active immunotherapy with T_reg_ depletion or functional blockade. Several studies showed that depletion of CD25^+^ cells *in vivo* in cancer patients could enhance the tumor-specific T cell responses induced by cancer vaccines (15). However, CD25-directed strategies may fail to achieve sustained results, since activated effector cells may be concomitantly eliminated and pT_reg_ may replenish the T_reg_ pool after depletion (15). Interestingly, a recent study has demonstrated that different T_reg_-depleting agents, either CD25-targeted (IL-2/diphtheria toxin fusion protein, or anti-CD25 antibody) or not (low-dose cyclophosphamide), failed to consistently eliminate more than 50% of committed T_reg_, as identified by TSDR demethylation (162).

Therefore, alternative strategies are needed to counteract the “hard core” of immune suppression that is represented by epigenetically committed T_reg_. We have proposed in the past that T_reg_ functional inactivation, rather than depletion, may represent a successful strategy to prevent massive pT_reg_ induction and concomitantly block T_reg_ suppression (15). This idea may be corroborated by the observation that markers associated to T_reg_ suppressive functions, and therapeutically targetable, may show enriched or restricted expression in epigenetically committed T_reg_. For instance, GITR stimulation has been shown to attenuate T_reg_ suppression and favor the rejection of experimental tumors (163). A recent study has demonstrated that GITR engagement *in vivo* led to the downregulation of Foxp3 expression in intratumoral T_reg_ (164). Of note, GITR^+^ T_reg_ were found enriched in Helios^+^ cells, representing highly committed T_reg_ (131), thus GITR targeting may preferentially block the strongest suppressors among the T_reg_ pool. A similar possibility could be envisaged for therapeutic strategies aimed at TNF-α/TNFR2 blockade, since this axis may be mainly involved in the activation of more committed and stable cells (122–126). Committed T_reg_ may also be targeted by virtue of their high proliferative potential: indeed, high proliferation rates, in terms of Ki67 positivity, were detected in healthy subjects within the aT_reg_ subset, enriched in stable and committed T_reg_ (117), and also in murine tumor-infiltrating Helios^+^ T_reg_ (131). Therefore, treatments based on the depletion of proliferating cells, such as low-dose cyclophosphamide, may efficiently target committed T_reg_.

An innovative way to improve immunotherapy would be to reprogram tumor-associated T_reg_ into fully armed effector cells, which would then become “exT_reg_.” Different from other vaccine-based approaches, T_reg_ reprograming is expected to trigger anti-tumor response very rapidly, since T_reg_ are already located at the tumor site and already tumor-antigen-experienced, thus not requiring a *de novo* T cell priming. Therefore, exT_reg_ may function in an “innate-like” manner, promptly providing co-stimulatory and pro-inflammatory signals when adequately modulated, before a novel adaptive anti-tumor response develops (140). An example of this approach has been proposed by Sharma et al. who demonstrated that reprograming of mature pre-existing tumor-associated T_reg_ into CD40L-expressing helper effector cells was needed to achieve tumor regression in a model of immunotherapy combining antigen vaccination, TLR-9 stimulation, and IDO blockade (152).

The above-discussed data overall indicate that tT_reg_ and pT_reg_ may not be equally susceptible to functional reprograming, but this dichotomy may turn into a benefit for the efficacy and safety of the evoked response. Indeed, on the one hand, tT_reg_, predominantly self-specific, highly committed, and hard to be reprogrammed into T-helper-like cells, would be preserved, thus ensuring immune tolerance to self-antigens and maintaining systemic immune homeostasis. On the other hand, pT_reg_, mainly representing tumor-specific and uncommitted cells, may be more easily converted into exT_reg_, thus mounting an immediate helper and/or effector response in a mostly tumor-antigen-specific fashion.

Reprograming into exT_reg_ may be achieved by immunotherapies aimed at subverting the immune suppression mechanisms established by innate cells in tumor microenvironments. For instance, in the above-reported model of tumor vaccination, CD40L upregulation by T_reg_ following TLR-9 stimulation was strictly dependent on host-derived MyD88 and IL-6 signals (152). In melanoma patients, tumor peptide antigen vaccination combined with low-dose cyclophosphamide and low-dose IL-2 evoked Th1-like T_reg_ accumulation, in line with a less tolerogenic microenvironment and with enhanced IL-12 availability (158). Of note, in this system, depletion of proliferating (conceivably committed and thymus-derived) T_reg_ by means of cyclophosphamide allowed the functional reshuffling of innate cells that in turn unveiled the emergence of Th1-like exT_reg_.

However, it is arguable that microenvironmental rearrangements would better accomplish full T_reg_ reprograming with the concomitant direct modulation of T_reg_ activities, aimed especially at enhancing T_reg_ susceptibility to external signals. For instance, expression of IL-12 receptor, which is epigenetically regulated in T_reg_ (135), could be artificially boosted by pharmacological approaches. Also, targeting with monoclonal antibodies some receptors expressed on T_reg_ surface and correlated with T_reg_ stability (such as TNFR2 and GITR) could result in enhancing T_reg_ propensity to reprograming. In line with this idea, treatment of murine melanomas with a GITR agonistic antibody resulted in the accumulation of exT_reg_ at the tumor site (164). Suppressor of cytokine signaling (SOCS) 1 and 2, which maintain Foxp3 stability and prevent T_reg_ polarization into effector cells (165, 166), may be pharmacologically inhibited to unlock T_reg_ responsiveness to pro-inflammatory microenvironmental cytokines.

## Conclusion

Even though discrimination between pT_reg_ and tT_reg_ by simple surface phenotyping is not yet possible many pieces of evidence indicate that both subsets contribute to the T_reg_ pool conditioning the tumor microenvironment. Nevertheless, the development/expansion of pT_reg_ and tT_reg_ are independent processes, possibly resulting from disparate antigens and signals, and their activities seem characterized by very peculiar features in terms of specificity, stability, and specialization. On the one side, tT_reg_ may expand at tumor site in response to self-antigens expressed by tumor cells, mostly include committed (TSDR-demethylated) Helios- and TNFR2-expressing cells, and contain the precursors of specialized T-bet^+^ Th1-suppressing cells, thus representing not only the guardians of systemic immune homeostasis but also the “hard core” of tumor immune escape. On the other side, pT_reg_ may mostly develop following local encounter with TAA or TSA antigens, possibly represent a mixed population of committed (TSDR-demethylated) and uncommitted (TSDR-methylated) cells, and are more prone to be reprogramed into Th1-like or Th17-like effector cells. We envisage that future successful immunotherapies may not only target committed T_reg_ but also favor “recycling” uncommitted T_reg_ into prompt anti-tumor effectors.

## Conflict of Interest Statement

The authors declare that the research was conducted in the absence of any commercial or financial relationships that could be construed as a potential conflict of interest.
